# Primary Cardiac Lymphoma Manifesting as Complete Heart Block

**DOI:** 10.1155/2020/3825312

**Published:** 2020-02-05

**Authors:** Courtney R. Usry, Andrew S. Wilson, Kelvin N. V. Bush

**Affiliations:** Division of Cardiology, San Antonio Military Medical Center, 3551 Roger Brooke Dr. San Antonio, TX 78234, USA

## Abstract

Primary cardiac tumors are exceedingly rare with variable clinical manifestations. This case involves a patient presentation of symptomatic complete heart block and cardiac imaging revealing a right atrial mass invading the myocardium consistent with Burkitt lymphoma on histopathology. The patient received definitive bradytherapy with a pacemaker and chemotherapy for the primary cardiac lymphoma. After three cycles of chemotherapy, the right atrial mass regressed significantly with restoration of atrioventricular conduction and no pacing burden. Primary cardiac lymphomas infrequently manifest as atrioventricular block and this case highlights cardiac masses as a potential etiology when evaluating new conduction disturbances and bradyarrhythmias.

## 1. Introduction

Primary cardiac tumors are exceedingly rare with a reported prevalence of 0.02% [1]. Primary cardiac lymphomas (PCL) are a subset of extranodal lymphomas where the primary tumor develops from the heart and/or the pericardium [2]. The incidence of PCL is 1-2% of primary cardiac tumors [3]. Secondary involvement of the heart is more common and reported to represent 16-28% of extracardiac lymphomas [4, 5]. Primary and secondary cardiac tumors are an important but often overlooked pathology to include in the differential diagnosis for patients presenting with bradyarrhythmias.

## 2. Case

**Figure 1:**
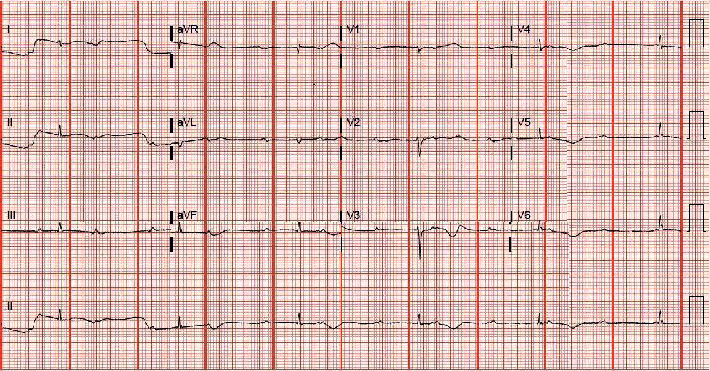
Electrocardiogram on presentation shows sinus rhythm, complete heart block with junctional escape rhythm.

**Figure 2:**
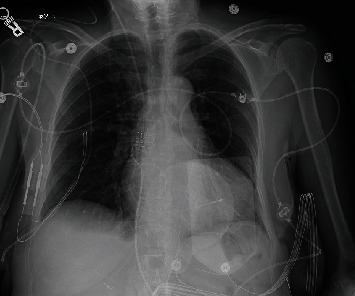
Chest X-ray single view with right jugular transvenous pacing wire with lead terminating in the right ventricle. There is a loop of the transvenous pacing wire seen within the right atrium.

**Figure 3:**
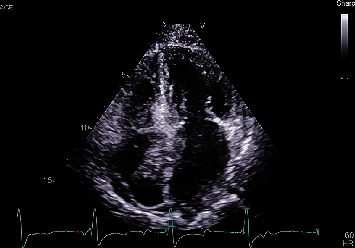
Apical four chamber from the transthoracic echocardiogram demonstrating a mass in the right atrium approximately 3-4 cm in size involving the interatrial septum.

**Figure 4:**
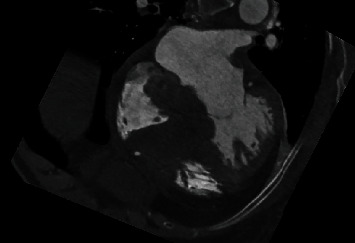
Four chamber view from the cardiac computed tomography angiogram demonstrating the mass invading the interatrial septum and inlet ventricular septum, and extending into the tricuspid annulus as well.

An 80-year-old high-functioning female with a history of hypertension presented to the emergency department for evaluation of new onset dyspnea. Vital signs were significant for a heart rate of 40 beats per minute in the absence of being treated with atrioventricular (AV) nodal blocking agents. Physical examination was remarkable for bradycardic heart sounds. The electrocardiogram (ECG) demonstrated sinus rhythm, complete heart block with a junctional escape rhythm (Figure 1). During admission, the junctional rhythm degraded to a ventricular escape rhythm and transvenous pacing was acquired via right internal jugular venous access. Chest X-ray showed looping of the lead in the right atrium while pacing the right ventricular apex (Figure 2). Transthoracic echocardiography revealed a hyperechoic dense structure in the right atrium (Figure 3). To further characterize the findings on echocardiography, additional imaging was pursued. Cardiac magnetic resonance imaging (MRI) from a multidisciplinary discussion was deemed to not be safe due to the presence of the temporary transvenous pacing wire. Therefore, cardiac computed topography angiography (CCTA) was performed and characterized the right atrial mass measuring 6.6 × 4.8 *cm* with Hounsfield units similar to myocardium without vascularization (Figure 4). The right atrial mass involved the tricuspid annulus, extending medially to the interatrial septum and inlet ventricular septum. Imaging of the coronaries, mediastinum, great vessels, abdomen, and osseous structures was noncontributory. Fluoroscopic-guided endomyocardial tissue biopsy of the mass was performed and revealed findings consistent with C-MYC positive, EBV-negative, Burkitt lymphoma with very high Ki-67 proliferation index (>95%), and positive for CD10 and CD20. The patient underwent implantation of a single ventricular lead pacemaker. For treatment of the PCL, systemic chemotherapy with rituximab plus cyclophosphamide, doxorubicin, vincristine, and prednisone (R-CHOP) was initiated. After three cycles of chemotherapy, native AV conduction was recovered with no pacing burden and the right atrial mass was decreased in size to 1 × 1 *cm* on repeat echocardiogram.

## 3. Discussion

PCL usually presents after the fifth decade of life and most often involves the right heart chambers [6]. Heart failure and pericardial effusions are the two most commonly reported clinical presentations of PCL [4]. AV block and ventricular tachycardia account for approximately 27% of the clinical manifestations of PCL and are secondary to direct infiltration of the conduction system or by irritation of the myocardium itself [7–9]. The CCTA in this case was instrumental in showing invasion of the atrial septum and inlet ventricular septum by the PCL.

The incidence of PCL has been on the rise in association with acquired immunodeficiency syndromes and transplant recipients receiving immunosuppressive therapy [9]. PCL has been a rarely reported malignancy in immunocompetent individuals and is often not diagnosed until there are advanced clinical manifestations [10]. A cardiac neoplasm was not suspected as the cause for our immunocompetent patient's complete heart block until imaging was obtained.

Echocardiographic findings concerning PCL or tumor invasions are recommended to be supported with a complementary imaging technique, either cardiac CT or MRI [11]. Cardiac MRI is a superior imaging modality with a higher sensitivity when compared to cardiac CT in identifying PCL, 90% versus 73%, respectively [8]. The presence of the temporary pacing wire excluded safe use of cardiac MRI in this case due to the risk of significant heating or dislodgement.

Development of various arrhythmias during chemotherapy has been described and highlights the importance of ongoing arrhythmia surveillance as the tumor substrate changes with treatment [6]. Other reported cases of AV block secondary to PCL with subsequent pacemaker implantation have reported resolution of heart blocks after initiation of chemotherapy without continued pacing requirements [4].

In the majority of patients with PCL, chemotherapy with CHOP has reigned as the treatment of choice [12, 13]. Adjunctive use of rituximab with CHOP for patients with B-cell lymphoma as described by Coiffier et al. has proven higher complete response rates in comparison to those who received CHOP alone, 76% versus 63%, respectively [7, 14]. Risk of death secondary to tissue necrosis can occur after the first cycle of chemotherapy, particularly in patients with significant myocardial infiltration [12]. For refractory cases of PCL, autologous stem cell transplantation and radiotherapy has had promising outcomes in few described cases [7, 15, 16].

Research by Dunleavy et al. has suggested that DA-EPOCH-R (dose-adjusted etoposide, prednisone, vincristine, cyclophosphamide, doxorubicin, and rituximab) may be a useful option in low-risk adult patients with Burkitt lymphoma. Additionally, in frail elderly patients with advanced Burkitt lymphoma, this regimen may be considered as an alternative treatment in an effort to reduce toxicity [17]. Additional studies are necessary to examine responses in biologic subtypes and risk subgroups to determine the optimal chemotherapy regimen to be utilized upfront [18–20]. Our patient had return of preserved AV conduction with R-CHOP, regression of the cardiac mass, and tolerated chemotherapy without subsequent cardiotoxicity.

Malignant cardiac tumors should be considered in the differential diagnosis when evaluating new conduction abnormalities or bradyarrhythmias. This case describes an immunocompetent patient presenting with complete heart block as the main clinical manifestation of a PCL. Early imaging and prompt histopathologic diagnosis is essential in diagnosing PCL, directing chemotherapy, and alleviating burden of AV block.
